# Anti-inflammatory effect of *Allium hookeri* on carrageenan-induced air pouch mouse model

**DOI:** 10.1371/journal.pone.0190305

**Published:** 2017-12-27

**Authors:** Jung-Eun Kim, Kyung-Mok Park, Soon-Young Lee, Ji-Hye Seo, In-Soo Yoon, Chun-Sik Bae, Jin-Cheol Yoo, Mi-Ae Bang, Seung-Sik Cho, Dae-Hun Park

**Affiliations:** 1 Department of Pharmacy, College of Pharmacy, Mokpo National University, Muan, Jeonnam, Korea; 2 Department of Pharmaceutical Engineering, Dongshin University, Naju, Jeonnam, Korea; 3 Department of Nursing, Dongshin University, Naju, Jeonnam, Korea; 4 College of Veterinary Medicine, Chonnam National University, Gwangju, Korea; 5 Department of Pharmacy, College of Pharmacy, Chosun University, Gwangju, Korea; 6 Research Development Team, Jeonnam Biofood Technology Center, Naju, Korea; Cairo University Faculty of Pharmacy, EGYPT

## Abstract

Inflammation is a commonly observed immune reaction, and rheumatoid arthritis is a particularly severe inflammatory disease. In this study, we used an air pouch mouse model to evaluate the anti-inflammatory potential of *Allium hookeri*, which has both been used as a culinary material and a traditional medicine in south-eastern Asia for many years. *Allium hookeri* suppressed typical symptoms of inflammation, such as condensation of the air pouch membrane, and inhibited the expression of several inducible proinflammatory cytokines such as IL-1β, IL-6, IL-13, and TNF-α. In order to determine the molecules modulating the inflammatory effect of carrageenan treatment, the components in *Allium hookeri* were analyzed by GC-MS, and linoleic acid, which have anti-inflammatory effect, was detected. From the results, we concluded that the anti-inflammatory effect of *Allium hookeri* might be attributed to linoleic acid, which could be promising candidates for anti-inflammatory drugs that have no adverse effects.

## Introduction

Inflammation is the host response to stimuli and is different from infections. In 2010 El-Gabalawy H *et al*.[1] reported that the prevalence of immune-mediated inflammatory diseases in Western countries reached 5–7%, and there are a lot of inflammatory disorders such as arthritis, asthma, atherosclerosis, colitis, dermatitis, and hepatitis. [2] In 2015, the Canadian Agency for Drugs and Technologies in Health (CADTH) reported that about 1% of Canadians were rheumatoid arthritis patients. [3]

There are many inflammation-related cytokines, although all cytokines cannot be clearly distinguished based on their functions, which include the induction or inhibition, stimulation and/or suppression of inflammation. They are only expressed under abnormal conditions (pathological states such as during a foreign body’s invasion) and they could induce at the invasion site molecules that alarm the body to the emergency situation. Interleukin (IL)-1β, IL-6, IL-13, and tumor necrosis factor (TNF)-α are inducible proinflammatory cytokines that are related to the initiation and propagation of inflammation. [4–8]

*Allium hookeri* (*A*. *hookeri*) has not only been used as a culinary material but also for traditional medicine in the south-east Asian region for many years. Various studies have established its bioactivities in terms of immunomodulation and prevention against microbial infection, coronary thrombosis and atherosclerosis. [9–13] The major components in *Allium* species include steroidal saponins, organosulfur compounds, etc. [14,15] *Allium* species have been reported to have antimicrobial and anti-inflammatory activities. [16–19] However, to the best of our knowledge, there has been no study on the chemical composition, anti-inflammatory activities of an ethanolic extract of *A*. *hookeri*. We identified the components of *A*. *hookeri* by GC/MS, and investigated its anti-inflammatory activity using mouse model of inflammation (carrageenan induced subcutaneous air pouch). In addition, this study also provides scientific information for further exploration and applications of this plant.

## Results

### 2.1. *A*. *hookeri* suppressed the proliferation of inflammatory cells in carrageenan-induced air pouch model

**Fig 1:**
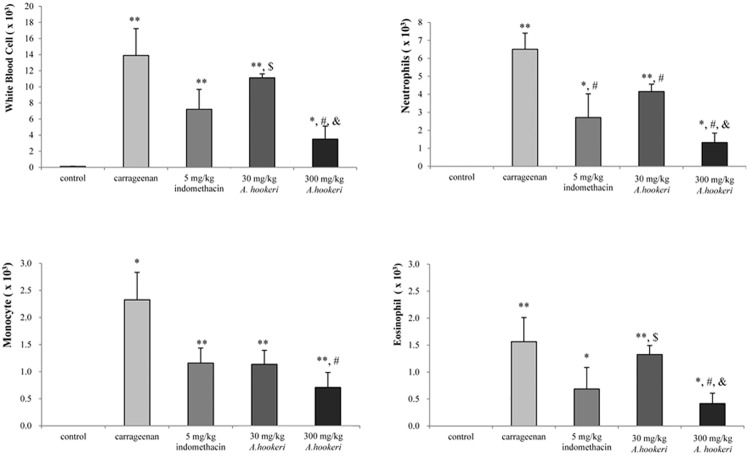
*Allium hookeri* suppresses the inflammatory cells proliferation in carrageenan-induced air pouch model. Animals were orally administered with different doses of *A*. *hookeri* 24 h and 1 h prior to carrageenan (1%) injection into the air pouch. The results are presented as mean ± S.D. (n = 9) of total leukocytes (× 10^3^ cells/mL). * vs. control, p < 0.05; ** vs. control, p < 0.001; $ vs. 5 mg/kg indomethacin treated group, p < 0.05; # vs. carrageenan treated group, p < 0.05; & vs. 30 mg/kg *A*. *hookeri* treated group, p < 0.05.

In order to evaluate the anti-inflammatory effect of *A*. *hookeri*, an air pouch mouse model was used and we first measured the change in inflammation-related blood cells in the exudate (Fig 1). The number of white blood cells (WBC) in exudates from the carrageenan-treated group increased by about 28 folds (14 × 10^3^ cells/mL) than that of the control group (0.5 × 10^3^ cells/mL) while that of the 5 mg/kg indomethacin-treated group decreased to 7 × 10^3^ cells/mL. *A*. *hookeri* dose-dependently suppressed the population of WBCs, for instance the WBCs level in the 300 mg/kg *A*. *hookeri*-treated group was less than that in the 5 mg/kg indomethacin-treated group. Furthermore, *A*. *hookeri* significantly reduced neutrophil, eosinophil, and monocyte proliferations induced by the injection of carrageenan, with suppression percentages of 80 ± 8 and 74 ± 12%, respectively.

### 2.2. *A*. *hookeri* prevented the carrageenan-induced morphological changes

**Fig 2:**
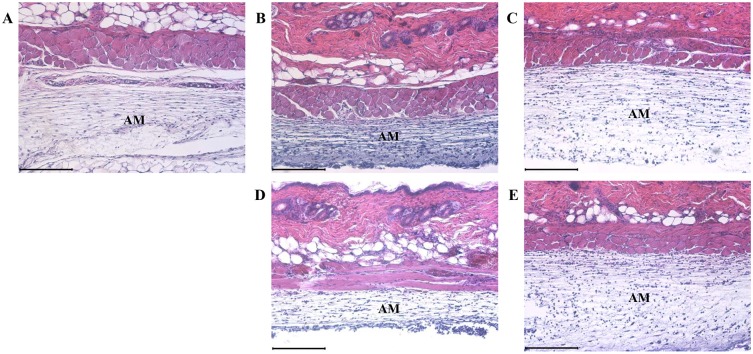
*Allium hookeri* suppresses the histopathological changes which are induced by carrageenan treatment. a, control; b, 1% carrageenan treated group; c, 5 mg/kg indomethacin treated group; d, 30 mg/kg *A*. *hookeri* treated group; e, 300 mg/kg *A*. *hookeri* treated group, AM, air pouch membrane; Scare bar, 200 μm.

In the skin, typical morphological changes resulting from carrageenan treatment-induced inflammation includes changes in the thickness of the air pouch membrane (Fig 2). In the carrageenan-treated group, the air pouch membrane was thin and condensed than in the control group. The thickness of the air pouch membrane in the indomethacin-treated group increased than that of the carrageenan-treated group. In the *A*. *hookeri*-treated groups, the thickness of the air pouch membrane dose-dependently increased while the thickness of the air pouch membrane in the 300 mg/kg *A*. *hookeri*-treated group was similar to that in the 5% indomethacin -treated group.

### 2.3. *A*. *hookeri* modulated the expression of inflammatory cytokines such as IL-1β, IL-6, IL-13, and TNF-α which was up-regulated in carrageenan-induced air pouch model

**Fig 3:**
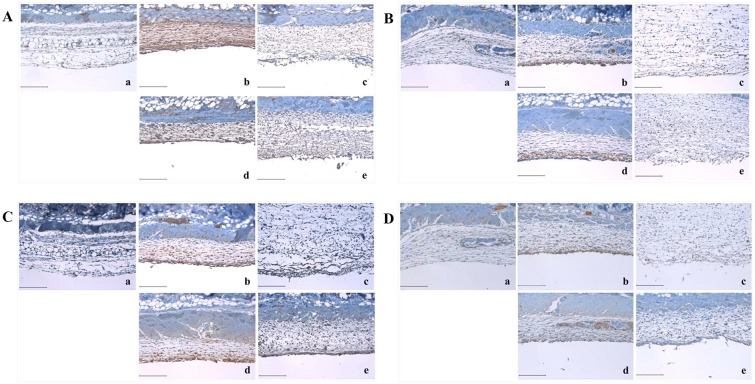
*Allium hookeri* suppresses the expression of several inflammation induction markers in the skin which are upregulated by carrageenan treatment. A is the photo of IL-1β expression, B is the photo of IL-6 expression, C is the photo of IL-13 expression, and D is the photo of TNF-α expression in the skin. a, control group; b, 1% carrageenan treated group; c, 5 mg/kg indomethacin treated group; d, 30 mg/kg *A*. *hookeri* treated group; e, 300 mg/kg *A*. *hookeri* treated group. Scare bar, 200 μm.

IL-1β in the carrageenan-treated group was significantly expressed compared to that of the control group (Fig 3Aa), which is shown in whole sites of the air pouch membrane (Fig 3Ab). Indomethacin effectively suppressed the level of IL-1β expression in the air pouch membrane (Fig 3Ac) and *A*. *hookeri* dose-dependently decreased IL-1β expression (Fig 3Ad and 3Ae). Similar to the results of IL-1β, carrageenan-induced expression of IL-6 increased in the margin of the air pouch membrane (Fig 3Bb) while indomethacin significantly inhibited the expression levels of IL-6 (Fig 3Bc), albeit in the 300 mg/kg *A*. *hookeri*-treated group, the expression levels of IL-6 was similar to that of the indomethacin-treated group (Fig 3Be). The change in IL-13 expression levels was the same as that of IL-6 (Fig 3C). Particularly, the expression level of IL-13 in the 300 mg/kg *A*. *hookeri*-treated group (Fig 3Ce) was almost similar to that in the indomethacin-treated group (Fig 3Cc). The level of TNF-α was increased by carrageenan treatment (Fig 3Db), but those in the *A*. *hookeri*-treated groups were dose-dependently suppressed (Fig 3Dd and 3De).

It was different from the distribution of IL-1β (Fig 3Ab) in the air pouch membrane, which was induced by carrageenan, and those of the other cytokines (Fig 3Bb, 3Cb and 3Db). IL-1β was expressed in the whole air pouch membrane area but IL-6, IL-13, or TNF-α was found around the margin of the air pouch membrane.

### 2.4. Linoleic acid which was analyzed in *A*. *hookeri* was suggested as an anti-inflammatory component

Linoleic acids (methyl ester) in *A*. *hookeri* were analyzed by GC/MS. GC/MS scanned mass fragmentations typically up to *m/z* 550 based on operation conditions (Table 1). Linoleic acids (methyl ester) were major components in *A*. *hookeri* and were typically characterized by its base peak at *m/z* 73, 337 (Linoleic acid) in mass fragment-grams (S1 Fig). A lot of peaks were found and through the similarity analysis compared to NIST library (2008 edition) about 500 compounds were estimated. However based on the percentage of them 52 compounds were listed (Table 2). Especially the mass spectrum of linoleic acids (methyl ester) in *A*. *hookeri* exhibited a molecular ion (*m/z* 337) in fragment with broad peaks between 28.3 min and 28.4 min during analysis in lapse time. The identified fragment-grams were compared to the organic standard and the peak could be easily identified by not only mass fragment-grams but also by mass spectrum in a NIST library (2008 edition). This summary is important in understanding the base level of relative mass amounts using m/z analytical approach related to reaction time as the major compound in *A*. *hookeri*.

**Table 1:** Analyzed compounds from *Allium hookeri* by GC/MS.

NO	RT (min)	Compound name	Quality	MolecularWeight (amu)	Content (%)
1	14.826	Benzene, 1-butenyl-, (E)-	96	132.094	0.01
2	15.466	Benzene, pentyl-	95	148.125	0.11
3	17.093	Benzoic acid trimethylsilyl ester	95	194.076	0.02
4	17.310	Benzene, hexyl-	94	162.141	0.04
5	18.406	Cyclohexasiloxane, dodecamethyl-	90	444.113	0.14
6	18.937	Nonanoic acid, trimethylsilyl ester	98	230.170	0.03
7	20.130	2,2'-Bipyridine	96	156.069	0.29
8	20.488	2,5-Cyclohexadiene-1,4-dione, 2,6-bis(1,1-dimethylethyl)-	96	220.146	0.04
9	21.714	Silane, (dodecyloxy)trimethyl-	97	258.238	0.22
10	22.245	2-Piperidinone, N-[4-bromo-n-butyl]-	91	233.042	0.17
11	22.755	Cyclopentadecane	95	210.235	0.14
12	22.820	Hexadecane, 2-methyl-	97	240.282	0.15
13	23.102	2,6-Diisopropylnaphthalene	95	212.157	2.03
14	23.243	Heptadecane	98	240.282	0.33
15	23.384	4-(3-Hydroxy-2,6,6-trimethylcyclohex-1-enyl)pent-3-en-2-one	90	222.162	0.98
16	23.634	1H-Indene, 2,3-dihydro-1,1,3-trimethyl-3-phenyl-	97	236.157	2.36
17	23.959	1,1'-Biphenyl, 2,2',5,5'-tetramethyl-	96	210.141	0.43
18	24.014	Hexadecane, 1-iodo-	94	352.163	0.16
19	24.328	1,4-Benzenedicarboxylic acid, bis(trimethylsilyl) ester	96	310.106	0.49
20	24.513	1-Eicosene	93	280.313	2.65
21	24.838	2-Phenanthrylamine, 9,10-dihydro-3,7-dinitro-	90	285.075	0.21
22	24.914	1-Hexacosene	94	364.407	1.04
23	25.055	Octadecane, 3-methyl-	91	268.313	0.14
24	25.294	Ethyl 13-methyl-tetradecanoate	93	270.256	0.43
25	25.511	9-Hexadecenoic acid, methyl ester, (Z)-	95	268.240	0.26
26	25.619	Hexadecanoic acid, methyl ester	97	270.256	0.39
27	26.237	Ethyl 9-hexadecenoate	99	282.256	1.95
28	26.400	Hexadecanoic acid, ethyl ester	99	284.272	3.27
29	26.552	1,2-Benzisothiazole, 3-(hexahydro-1H-azepin-1-yl)-, 1,1-dioxide	91	264.093	0.13
30	26.671	Cyclotetradecane, 1,7,11-trimethyl-4-(1-methylethyl)-	93	280.313	0.11
31	26.715	cis-9-Hexadecenoic acid, trimethylsilyl ester	95	326.264	0.21
32	26.801	Hexadecanoic acid, trimethylsilyl ester	99	328.280	0.58
33	26.932	Pentacosane	94	352.407	0.27
34	27.268	9,12-Octadecadienoic acid (Z,Z)-, methyl ester	99	294.256	1.80
35	27.669	Linoleic acid ethyl ester	99	308.272	8.02
36	28.179	Octadecanoic acid, ethyl ester	98	312.303	0.76
37	28.222	Docosane	98	310.360	3.21
38	28.515	Eicosane	94	282.329	0.34
39	28.580	Octadecanoic acid, trimethylsilyl ester	99	356.311	0.44
40	28.765	Tributyl acetylcitrate	90	402.225	0.54
41	29.047	Tetratriacontane	91	478.548	0.93
42	29.123	Heneicosane	93	296.344	0.36
43	29.350	2-Dodecen-1-yl(-)succinic anhydride	92	266.188	0.31
44	30.457	Dodecanoic acid, undecyl ester	93	354.350	0.78
45	30.533	Tricosane	91	324.376	3.73
46	30.739	Tetracosane	95	338.391	0.85
47	30.869	24-Norcholane, 23-[2-methyl-1-(1-methylethyl)cyclopropyl]-, (5.alpha.)-	92	412.407	2.44
48	30.945	Hexacosane	94	366.423	7.50
49	31.173	Bis(2-ethylhexyl) phthalate	91	390.277	0.85
50	33.028	l-Methionine, N-neopentyloxycarbonyl-, tetradecyl ester	90	459.338	0.40
51	34.818	Nonacosane	95	408.470	0.99
52	35.631	2-Ethoxycarbonyl-3-methyl-4-azafluorenone, 2-fluorenylimime	90	430.168	0.29
53	35.750	.alpha.-Tocopherol (vitamin E), trimethysilyl derivative	96	502.421	1.09
54	36.152	Cholestan-3-one, (5.alpha.)-	96	386.355	0.42
55	36.249	Z-14-Nonacosane	94	406.454	0.40
56	38.191	.beta.-Sitosterol trimethylsilyl ether	97	486.426	0.49
57	38.527	.alpha.-Tocopherol (vitamin E), trimethysilyl derivative	98	502.421	1.24
58	39.818	Stigmast-4-en-3-one	96	412.371	0.79

**Table 2:** Operation parameters for the GC/MS. [1] HP Agilent GC:7890A MS:5975C.

Condition	GC / MS [1]			
Column	J&W Scientific, DB-5 cross linked 5% phenylmethyl silicone			
Carrier	Helium			
Split / Splitless	Splitless			
Injection Volume	1.0 μL			
Detector	MS			
MS Source	230°C			
MS Quad	150°C			
Analytical Temperature		Rate (°C/min)	Value (°C)	Hold time (min)
	initial	-	65	10
	Step 1	10	300	22
	Total	55.5 min		
Electron Ionization	70 ev			
Mass Range	50–550 amu			
Scan method	Full Scan			

## Discussion

IL-1 consists of IL-1α and IL-1β. IL-1β is associated with various inflammatory diseases such as rheumatoid arthritis, auto-inflammatory diseases, etc. and IL-1β neutralization is mostly used for inhibiting inflammation. [5] IL-6 expression is increased by inflammatory stimuli and is subsequently spread into the whole body. [4] IL-13 and TNF-α play important role in inflammation and their levels increase in rheumatoid arthritis. [6,7] Carrageenan air pouch is a particularly good model for evaluating the relationship of inflammation and other factors such as cytokines, prostaglandins, etc. TNF-α plays an important role in inducing inflammation in air pouch models [8] and is one of the mediators that play an important role in the inflammatory process; they are released from activated monocytes and macrophages and cause increased vascular endothelial permeability. [9]

In this study the level of four inducible proinflammatory cytokines such as IL-1β, IL-6, IL-13, and TNF-α was increased by carrageenan treatment, albeit *A*. *hookeri* could suppress their levels irrespective of their different expression sites.

Carrageenan air pouch models are usually used for both acute and chronic inflammation studies due to the ease in setting it up and interpreting the results. [20] The air pouch model is one of the most helpful experiments used to determine the effects of anti-arthritic agents, as the air pouch lining has been reported to display a histological similarity to synovial membranes. [21] Therefore, we can hypothesize that the root extract of *A*. *hookeri* might be a good anti-inflammatory food for arthritis. However, the effect of the root extract of *A*. *hookeri* in a more specific animal model of arthritis remains to be assessed in future studies. Nine compounds which content was 2.0% or more were analyzed such as linoleic acid ethyl ester (8.02%), hexacosane (7.5%), tricosane (3.73%), hexadecanoic acid ethyl ester (3.27%), docosane (3.21%), 1-eicosene (2.65%), 24-norcholane (2.44%), 1H-indene, 2,3-dihydro-1,1,3-trimethyl-3-phenyl- (2.36%), and 2,6-diisopropylnaphthalene (2.03%) but most of all were fatty acids that don’t have anti-inflammatory effect. However linoleic acid is a fatty acid that exists in nuts, vegetable oils, etc. [22] It has various effects on organisms such as antioxidant [23] and anti-inflammatory [24] effects. Although there are many elements associated with the anti-inflammatory activity of *A*. *hookeri*, linoleic acid could be considered effective component as their anti-inflammatory effects have been elucidated. Additionally, the content of hexacosane (7.5%) was similar to that of linoleic acid (8.02%). hexacosane has been reported to have antimicrobial activity against infectious bacteria such as against *Klebsiela pneumoniae*, *Salmonela typhi*, *Stapylococcus aureus*, and *Proteus vulgaris*. [25] Therefore, hexacosane of *A*. *hookeri* is thought to contribute to inflammation related to the infectious diseases by antimicrobial effect.

The present study shows that *A*. *hookeri* possesses anti-inflammatory subtance. One phytochemical identified from the *A*. *hookeri* might be responsible for its biological activities. This study provides a good basis for future development of *A*. *hookeri*-based medicinal preparations as a preventive source. In the future, we will purify the other effectors and test whether these effectors has beneficial effect on other inflammatory diseases such as arthritis.

The results of the present study confirm and advance those of earlier reports, which have demonstrated *in vitro* the anti-inflammatory activity of root extracts of *A*. *hookeri* [26] and demonstrated for the first time the marked anti-inflammatory effects of *A*. *hookeri* root extract. From the results we concluded that it is possible that *A*. *hookeri* is an anti-inflammatory drug candidate.

## Materials and methods

### 4.1. Plant material and 70% EtOH extract preparation

*A*. *hookeri* was collected on May 2014 near the Naju city, Jeonnam province in Korea. A voucher specimen (MNUCSS-SC-01) was deposited at the College of Pharmacy, Mokpo National University. The root was separated for the study. Air-dried, powdered *A*. *hookeri* root*s* (250 g) *were* extracted twice with 70% ethanol (1 L) at room temperature for three days. After filtration, the ethanol was evaporated, freeze dried and stored at −50°C. The crude extract was re-suspended in distilled water and filtered using a 0.4 μm membrane.

### 4.2. Carrageenan-induced inflammatory air pouch mouse model

Male ICR mice (20–25 g) were purchased from Orient Bio Inc. (Seongnam, Korea). In order to make space (or air pouch) under the skin, 2 mL of air was injected thrice into the intra-scapular area of the mice’s back for six days. The animals were subsequently divided into two categories of which one (n = 6) was not treated with carrageenan while the other (n = 24) was treated with carrageenan; the animal study was undertaken twice using the same protocol. The carrageenan-treated category consisted of four groups including an inflammation-induced group that had been injected with carrageenan after normal saline oral administration, a 5 mg/kg indomethacin-treated group (used as an anti-inflammatory drug), a 30 mg/kg *A*. *hookeri*-treated group, and a 300 mg/kg *A*. *hookeri*-treated group. Indomethacin and *A*. *hookeri* were orally administered. At two hours after treatment, the carrageenan solution (1 mL, 2 w/v % dissolved in saline) was injected into the air pouch and after 24 h of carrageenan injection, all mice were sacrificed using with over-dosing of Zoletil (Virbac, Carros, France) via intraperitoneal injection. To collect the exudate, the pouches were flushed with 2 mL of phosphate buffered saline (PBS). The number of total and differential cells in the exudate from the pouch was counted using the Hemavet Multispecies Hematology System (Drew Scientific Inc., Waterbury, CT, USA).

### 4.3. Histopathologic analysis

After harvesting the exudate, the skin tissues were collected, fixed in 10% (v/v) formaldehyde solution, dehydrated in a graded ethanol series (99.9%, 90%, 80%, and 70%), and embedded in paraffin. Paraffin-embedded skin tissues were then sectioned (4 μm) and stained with hematoxylin and eosin.

### 4.4. Immunohistochemical analysis

Deparaffinized tissue sections were treated with 3% hydrogen peroxide in methanol for 10 min to remove endogenous peroxidase. Antigen retrieval was carried out with sodium citrate buffer (0.1 M) using the boiling method. The slides were incubated with normal serum to block nonspecific binding and then incubated overnight at 4°C with the following primary antibodies (diluted 1:100 or 1:200): IL-1β (Santa Cruz, CA, USA, sc-1251), IL-6 (Santa Cruz, sc-7920), IL-13 (Santa Cruz, sc-1776), IFN-γ (Santa Cruz, sc-74104) and TNF-α (MY BioSource, CA, USA, MBS175453). The slides were incubated for 2 h with biotinylated secondary antibody (1:500; DAKO, Carpinteria, CA, USA) and horseradish-peroxidase conjugated streptavidin. Signals were detected using 3,3-diaminobenzidine tetrahydrochloride substrate chromogen solution, and cells were counterstained with Mayer’s hematoxylin.

### 4.5. Ethics statement

All animals were maintained according to the guidelines of the Institutional Animal Care and Use Committee (IACUC) in Dongshin University and the study had acquired an approval number (Approval No. 2014-08-03) from Dongshin University IACUC.

### 4.6. Gas chromatography mass spectrometry (GC-MS) analysis

The analytical methods for the organic analysis, based on gas chromatography mass spectrometry (GC-MS), have been previously reported. [27] Briefly, GC-MS (HP Agilent GC: 7890A MS:5975C, Agilent Technologies, Santa Clara, CA, USA) was tuned by perfluorotributylamine (PFTBA) using three mass fragments (m/z) of 69.0, 219.0, 502.0 in the condition of electron ionization (EI). A 5MS GC column (DB-5 cross-linked 5% phenylmethyl silicone, J&W Scientific, Agilent Technologies) was used for the analysis as this column has low bleed that improves sensitivity for constituent identification. The GC oven was heated using the following conditions: isothermal at 65°C for 10 min and 10°Cmin^-1^ to 300°C with helium (He) as carrier gas. The summarized operation parameters for the GC-MS are shown in Table 1.

In order to analyze the quality of the *A*. *hookeri* extract, certain amounts of dried samples, using dehydrofreezing procedure, was prepared. The samples were extracted twice with dichloromethane followed by additional sonication (twice) with acetone using mild sonication at ambient temperature for 10 min. The extract was evaporated using high volume nitrogen blowdown (TurboVap II, Caliper Life Sciences, Mountain View, CA, USA) to obtain 5–10 mL and was further evaporated to a final volume of 100 μL using low volume nitrogen blowdown (MGS-2200, Eyela, Tokyo Rikakikai Co., LTD, Tokyo, Japan). A final extract volume of 100 μL was silylated with N,O-bis(trimethylsilyl)trifluoroacetamide (SUPELCO, St. Louis, Mo, USA) to derivatize the constituents to their trimethylsilyl-derivatives (TMS-derivatives) before GC-MS analysis. [28] The silylated samples were all analyzed with GC-MS. A set of authentic quantification standard including linoleic acid was used for GC-MS identification and quantification.

Supplementary 1 presents the identification of silylated linoleic acids (methyl ester) using a standard solution. Linoleic acids (methyl ester) can be analyzed at retention time (RT) of 28.389, respectively. The m/z identification can be 73.1 & 337.3 for linoleic acid (methyl ester), which can be confirmed by the NIST mass spectral library (2008 Edition, National Institute of Standards and Technology, Gaithersburg, MD, USA).

### 4.7. Statistics

Results are expressed as mean ± standard deviation (SD). Group differences were evaluated by one-way analysis of variance, followed by Dunnett’s multiple comparison test. Significance was considered at *p* <0.05 or *p* <0.001.

## Supporting information

S1 Fig GC-MS chromatograms of whole extract and linoleic acid in *Allium hookeri*. (TIF)

S1 Graphical Abstract *Allium hookeri* suppressed the carrageenan-induced inflammation. *Allium hookeri* contains 8.02% linoleic acid which might be as an anti-inflammatory component and suppressed the inflammation via down-regulations of several cytokines such as IL-1β, IL-6, IL-13, and TNF-α.
(TIF)
