# Supplementary figures and images for: Anti-inflammatory effect of Allium hookeri on carrageenan-induced air pouch mouse model
Source: PLoS One. 2017 Dec 27;12(12):e0190305. doi: 10.1371/journal.pone.0190305 (PMC5744995; doi:10.1371/journal.pone.0190305)

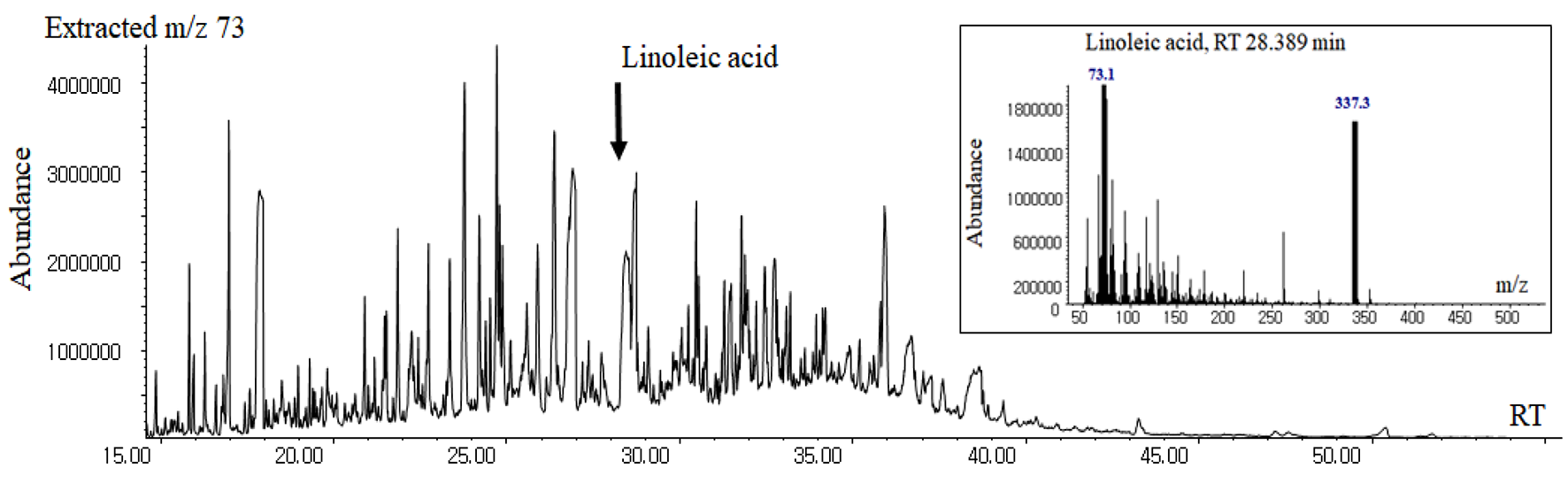

Supplement: S1 Fig — (TIF) [file pone.0190305.s001.tif]

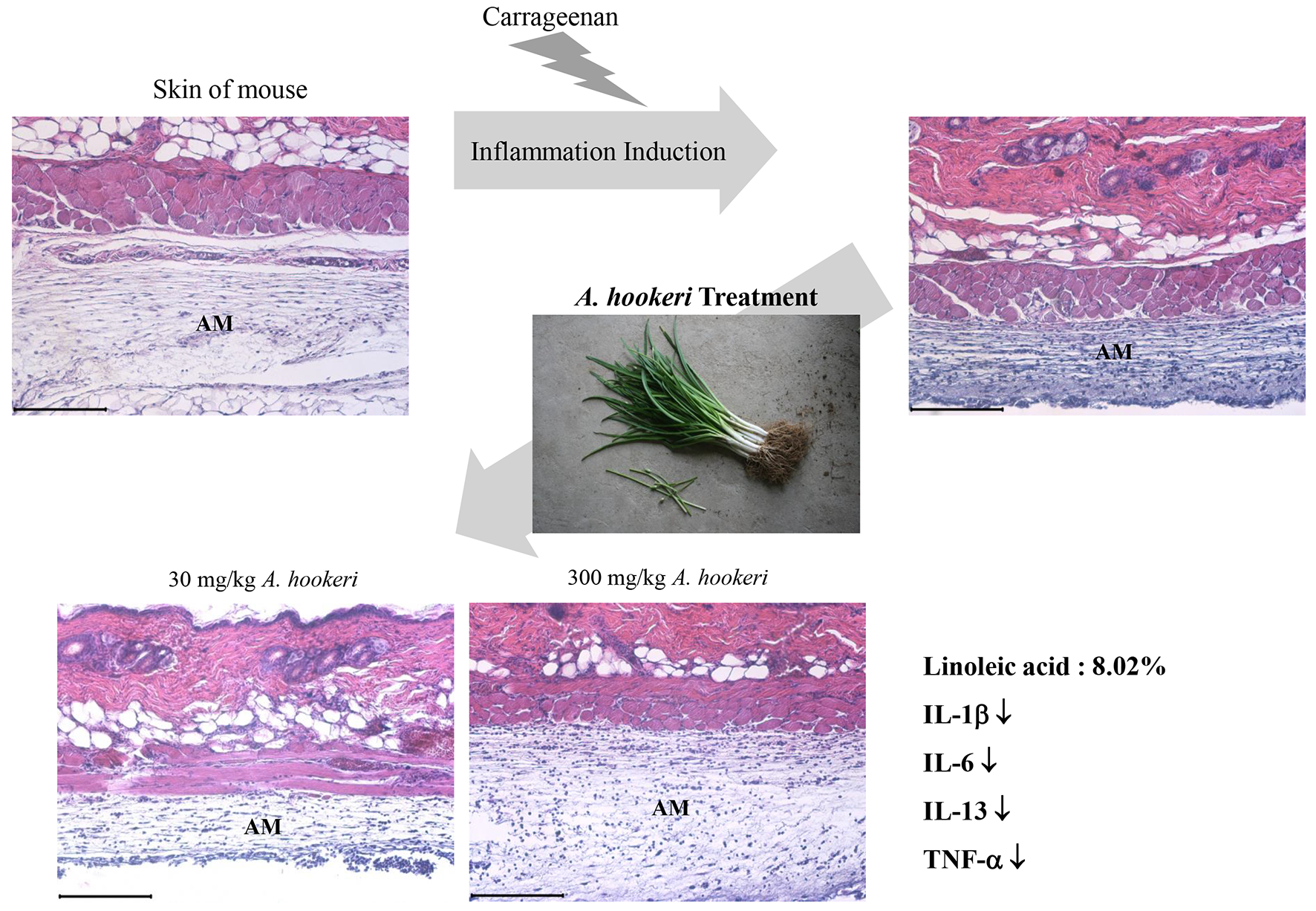

Supplement: S1 Graphical Abstract — Allium hookeri contains 8.02% linoleic acid which might be as an anti-inflammatory component and suppressed the inflammation via down-regulations of several cytokines such as IL-1β, IL-6, IL-13, and TNF-α. (TIF) [file pone.0190305.s002.TIF]
